# The English Plastic Bag Charge Changed Behavior and Increased Support for Other Charges to Reduce Plastic Waste

**DOI:** 10.3389/fpsyg.2019.00266

**Published:** 2019-02-26

**Authors:** Gregory Owen Thomas, Elena Sautkina, Wouter Poortinga, Emily Wolstenholme, Lorraine Whitmarsh

**Affiliations:** ^1^Welsh School of Architecture, Cardiff University, Cardiff, United Kingdom; ^2^National Research University Higher School of Economics, Moscow, Russia; ^3^School of Psychology, Cardiff University, Cardiff, United Kingdom

**Keywords:** sustainability, behavior, attitudes, spillover, plastic, policy, bag charge

## Abstract

Plastic bags create large amounts of waste and cause lasting environmental problems when inappropriately discarded. In 2015, England introduced a mandatory five pence (US$0.06/€0.06) charge to customers for each single-use plastic bag taken from large stores. Combining a longitudinal survey (*n* = 1,230), supermarket observations (*n* = 3,764), and a longitudinal interview study (*n* = 43), we investigated people’s behavioral and attitudinal responses to the charge. We show that all age, gender, and income groups in England substantially reduced their plastic bag usage within 1 month after the charge was introduced, with interviewees highlighting the ease of bringing their own bags. Support for the bag charge also increased among all key demographic groups. Increased support for the plastic bag charge in turn predicted greater support for other charges to reduce plastic waste, suggesting a “policy spillover” effect. Results indicate a broad and positive effect of the bag charge, which appears to have catalyzed wider waste awareness among the British public. This may facilitate the introduction of other policies to eliminate avoidable single-use plastics and packaging.

## Introduction

The single-use plastic carrier bag has become a common feature of modern shopping since their introduction in the 1980s. In 2014, over 8.5 billion plastic bags were used by United Kingdom supermarket shoppers, estimated to produce 58,000 metric tons of plastic waste (WRAP, 2015). Plastic bags mostly end up in landfill as part of the household waste stream, but can also cause severe damage to wildlife and clog drains and waterways when they end up in the environment (Barnes et al., 2009; Gregory, 2009; BIO Intelligence Service, 2011). As such, they represent a significant environmental and public health threat, and are also emblematic of broader sustainability challenges arising from increasing levels of consumption and waste. In response, national and local governments across the world have introduced legislation to reduce the environmental burden of plastic bags, including bans and mandatory charges (Miller, 2012). All four countries of the United Kingdom (UK) have now introduced a mandatory five pence (US$0.06/€0.06) charge to customers for each single-use plastic carrier bag issued by retailers: typically defined as bags with handles that are less than 70 microns thick and not designed for reuse (HM Government, 2015). Consumers’ behavioral and attitudinal responses to these policies have been dramatic and consistent. Retailers estimate that the usage of single-use plastic bags has fallen by about 80% in Wales, Northern Ireland, and Scotland since their introduction in 2011, 2013, and 2014, respectively (WRAP, 2015; zero Waste Scotland, 2015). Mandatory charges are not only effective in reducing plastic bag use, they also appear to be popular among the public. Support for a carrier bag charge in Wales was already high before it was introduced, and increased even further after (Poortinga et al., 2013). A similar bag charge introduced in the Republic of Ireland in 2002 has been described as “the most popular tax in Europe” (Convery et al., 2007). The mechanism of how a bag charge affects people is still unclear. Some view a bag charge as an economic instrument, where increasing the cost of a plastic bag alters the cost-benefit calculation, and discourages purchase of the item (Dikgang et al., 2012). Alternatively, bag charges have been suggested as a way of disrupting the automatic use of plastic bags by changing people’s typical bag-use routine (Poortinga et al., 2013; Jakovcevic et al., 2014).

Previous investigations into plastic bag charge policies vary in methodology but may not have captured a full range of behavioral and/or personal responses to such a policy. Economic-focused studies examined changes in behavior by observing bag use by shoppers in the field (Homonoff, 2013) or compared the volume of bags issued by supermarkets with different socio-economic profiles (Dikgang et al., 2012). Other investigations used pre and post-bag charge surveys in Wales to establish behavioral and attitude changes but used independent samples (Poortinga et al., 2013), or analyzed longitudinal secondary data with broad measurements that may not capture specific responses linked to the bag charge (Thomas et al., 2016). Additional research on a plastic bag charge in Argentina successfully combined observations and brief survey measurements, but without longitudinal comparisons (Jakovcevic et al., 2014), and we are not aware of any evidence based on qualitative data within bag charge policy studies. Here, we offer the first longitudinal analysis of how a national bag charge policy affects individuals experiencing the charge, and draw upon a range of methodologies to evaluate views and behavior at a personal and aggregate level.

The success of behavior change policies for sustainable outcomes is dependent on public support. However, little is known about how different groups respond to such policies and whether they might inadvertently exacerbate social sustainability problems while addressing environmental ones. As a flat fee, a bag charge may have a more profound effect on lower-income households, potentially leading to greater behavior change but lower levels of support. Conversely, a small charge could lead to less behavior change among higher-income households (Ayalon et al., 2009; Dikgang et al., 2012; Fairhead, 2015). Furthermore, older age groups are the most likely, and young men are the least likely, to use reusable bags for shopping (Homonoff, 2013; WRAP, 2014). While there is greater potential for behavior change among the latter group, the impact of a plastic bag charge on different socio-demographic groups remains uncertain. Among concerns of unfair application of a flat fee upon the population, it is worth considering how support for a bag charge after implementation varies among various demographic groups.

In terms of attitudinal responses, there is some evidence that people become more supportive of a bag charge after it is introduced (Poortinga et al., 2013). This effect has also been observed for other environmental, safety, and health policies. For example, Nilsson et al. (2016) showed that attitudes toward a congestion tax became more positive after its implementation in Gothenburg, Sweden; Fong et al. (2006) found increases in support for smoke-free public places following the implementation of comprehensive smoke-free workplace legislation in the Republic of Ireland; and Dinh-Zarr et al. (2001) reported that the public have increasingly positive attitudes toward enhanced safety belt enforcement programs. This raises some interesting questions about the role of public attitudes when implementing policy measures. It is also suggestive of attitudes following behavior and behavior change, as suggested by Cognitive Dissonance Theory (Festinger, 1957).

Beyond the primary effect of the charge on behavior and attitudes, it is also beneficial to determine whether wider policy support effects may be observed. The phenomenon of “behavioral spillover” is one such example, broadly defined as the effect where change in one behavior causes a change in another separate but related behavior. There is now a comprehensive literature on behavioral spillover, summarized in recent reviews (see e.g., Truelove et al., 2014; Nash et al., 2017). Spillover research is primarily focused on behaviors, with examples of spillover found between purchasing sustainable goods and increased frequency of other sustainable actions (Lanzini and Thøgersen, 2014), an example of positive spillover where increases in one behavior are matched in another. But there is also scope for negative spillover, as reported by Thøgersen and Ölander (2003) where purchasing organic food predicted lower usage of public transport. Mechanisms of spillover remain unclear, but are generally viewed as a process that involves some internal changes, be it environmental goals or values, personal identity, self-efficacy, or skills and knowledge (Thøgersen, 2012). Indeed, spillover is not limited to behavior, but may also be linked to changes in personal views, such as support for environmental policies. Previous work highlighted the relationship between sustainable consumerism and support for sustainable policies (Thøgersen and Noblet, 2012), but experimental work suggests that engaging in sustainable behavior may generate negative spillover effects (reduced support for a “green fund”) among people more politically aligned to sustainable policies (Truelove et al., 2016). The introduction of plastic bag charges has generated several explorations of behavioral spillover, with previous investigations casting doubt upon a causal effect of charges and behavioral spillover (Poortinga et al., 2013; Thomas et al., 2016). The wider concept of *policy spillover* effects may play a role, however. Given the popularity of bag charges (Convery et al., 2007), additional sustainable policies may increase in popularity as a result of changed views on a plastic bag charge. That is, experiences with a policy may not only change public views regarding that particular policy, it may also change views regarding other. To date, we believe this is the first investigation to directly explore how introduction of a policy may cause spillover that would affect support for other, similar policies.

In October 2015, a plastic bag charge was introduced in England to reduce the use of avoidable single-use plastics. We conducted a multi-method, longitudinal and controlled investigation comprising three elements: (1) a longitudinal survey; (2) a longitudinal interview study; and (3) a longitudinal observational study. In all three elements, data from England were compared to Wales and/or Scotland to ensure that changes in attitudes and behavior cannot merely be attributed to some larger cultural shift in attitudes and/or other extraneous influences. At the time of the study, both Scotland and Wales had already introduced a charge on single-use carrier bags, and there were no known changes in the policy landscape that may have impacted on the results. The three methodological elements were combined to deliver a comprehensive, controlled and in-depth investigation of behavioral and attitudinal changes following the introduction of the charge, highlighting areas where the different methods converge, corroborate and complement each other (Johnson et al., 2007). This means that the results can be triangulated and validated using the different methodologies. In our study, the triangulation of survey findings with the observational data helped to corroborate the survey data and counteract the frequent biases of self-reports. In addition, the triangulated use of interview data not only helped corroborate survey and observational findings, but also gain a valuable in-depth insight into participants’ lived experiences of the processes of behavior and attitude change that accompanied the charge introduction. Finally, adding both interview and observational methods allowed us to show how the intervention (i.e., introduction of the plastic bag charge policy) was implemented in real world.

## Materials and Methods

The study used a mixed-methods longitudinal approach, and involved (1) a longitudinal survey; (2) a longitudinal interview study; and (3) a longitudinal observational study. All materials and data for the three elements are available under user license from the United Kingdom Data Service: http://reshare.ukdataservice.ac.uk/852642/.

### Longitudinal Survey

The longitudinal survey measured behavior and views from representative samples in England (*n* = 728), Wales (*n* = 271), and Scotland (*n* = 231) at three points: 1 month before (T1), 1 month after (T2), and 6 months after (T3) the English plastic bag charge was introduced. The longitudinal survey was approved by the Welsh School of Architecture Research Ethics Committee (EC1507.239). The survey was hosted by market research company Ipsos MORI, using their pre-established online access panel, with additional samples recruited in Wales to ensure representative coverage of all three countries. The survey was advertised as a household shopping behavior survey. Representative sampling quotas were set in all countries for age, gender and employment status, with employment status quotas based on Eurostat 2013, and other variables based on Eurostat 2012 data. Additional quotas for geographical region were set for England. The number of respondents completing the surveys at the three time points (T) is shown in Table 1.

**Table 1:** Number of respondents completing each survey by country of residence.

	T1	T2	T3
Country	September 2015	November 2015	April 2016
England	1,802	1,191	728
Wales	664	422	271
Scotland	600	392	231
Total	3,066	2,005	1,230

Retention rates between T1 and T3 for England (40.4%), Wales (40.8%), and Scotland (40.1%) were comparable, *X*^2^(2) = 0.85, *p* = 0.655. Additionally we found that attrition was not linked to any level of baseline support for the plastic bag charge, *X*^2^(4) = 2.76, *p* = 0.599.

**Shopping bag use** was measured in two ways. First, we asked the question “*How often, if at all, do you take a single-use plastic bag from the till* [point of purchase] *when doing your main food shop/top-up food shop?”*, with a five-point response scale ranging from 1 (Never) to 5 (Always), and a “don’t know” response coded as missing. Second, we asked “*How often, if at all, do you usually take your own shopping bag(s) to each of the following stores?”* with options for “*Food store for a main food shop*” and “*Food store for a top-up food shop*” measured using the same response scale.

**Public support for a bag charge** was assessed using one item: “*To what extent do you support or oppose a 5p charge to the customer for each single-use plastic bag used?*” using a five-point scale ranging from 1 (Strongly oppose) to 5 (Strongly support), with an additional option of “don’t know” coded as missing.

**Support for other charges to reduce plastic waste**: we presented two statements with hypothetical plastic waste reduction policies for people to indicate their support or opposition. The first statement read:

There have been some suggestions that because of the amount of plastic used in their manufacturing, there may be an additional charge of 5p added to the purchase of each plastic water bottle. To what extent would you support or oppose an additional charge of 5p for plastic bottles?

The second statement read:

There has also been some discussion that with the amount of plastic used today, there may be an additional charge of 5p added to products with a lot of plastic packaging, such as individually wrapped fruit or vegetables. To what extent would you support or oppose an additional charge of 5p for products with a lot of plastic packaging?

We presented a third statement discussing a fuel duty for people to indicate their support or opposition. This policy was included as a non-waste environmental measure, as a “control” measure for which we did not expect a policy spillover effect. The statement read:

To address the amount of emissions caused by burning motor fuel, there has been some discussion that the government may raise tax charged on petrol and diesel. To what extent would you support or oppose an increase in taxes charged on petrol and diesel?

People could indicate their opposition or support for the three policies on a five-point scale from 1 (Strongly oppose) to 5 (Strongly support), with an additional option of “don’t know” coded as missing.

Analysis of data was performed in IBM SPSS V.20. Analysis of changes in behavior and policy support were run using Linear Mixed Models (LMM), which allows for longitudinal analyses that can work with incomplete data sets without loss of statistical power. The LMMs applied an unstructured repeated covariance matrix, which allows for greater flexibility when calculating variance of data points and covariances between measurements without prior assumptions. When analyzing changes in behavior or policy support over time, the time of each survey measurement (“Time”) and country of respondent (“Country”) were specified as fixed factors, with an interaction term between Time and Country establishing if the dependent variable varied between countries over time. Analyses of changes in behavior and policy support used a similar approach, replacing the fixed factor of Country with “Gender” (coded 0 = Male and 1 = Female), “Age” (four groups of age brackets), and “Income” (four groups of income bracket).

### Longitudinal Interview Study

For the longitudinal interview study, we recruited respondents (*n* = 43) in England, Wales, and Scotland. Respondents were interviewed 1 month before (T1) and 1 month after (T2) the English bag charge was introduced. This was part of a larger methodological strategy using the diary-interview method (Zimmerman and Wieder, 1977). In this paper, we have chosen to present only the interview data because it provided the most in-depth information on behavior and attitude change. The interview study was approved by the Welsh School of Architecture Research Ethics Committee (EC1507.243). Interview participants lived in geographically diverse locations across England, Scotland, and Wales. An external company recruited participants who were broadly representative of gender, age, socioeconomic status, and urban/rural location across the three countries. In total, 14 participants in England, 13 in Scotland, and 16 in Wales were interviewed pre- and post-bag charge (Table 2).

**Table 2:** Sample sizes of the interview study.

	T1	T2
	September 2015	November 2015
England	18	14
Wales	18	16
Scotland	16	13
Total	52	43

The study aim was presented to participants as research on people’s household behaviors, procedure was explained in detail, and participants were guaranteed anonymity. Semi-structured in-depth interviews were conducted over the telephone by three authors (ES, EW, and GT), and lasted between 45 and 75 min. Interviews were digitally recorded with participants’ written informed consent and anonymised. Semi-structured interviews were designed to allow for an in-depth exploration of emerging themes as well as salient issues surrounding the processes of behavior and attitude change related to the English plastic bag charge. The interview topic guide included questions on shopping and bag use behaviors, attitudes to the plastic bag charge, attitudes to other environmental charges, environmental behaviors and attitudes, and socio-demographics.

Interview data was transcribed verbatim, and transcripts were checked against recorded audio-files. Transcripts were coded and thematic analysis (Miles and Huberman, 1994; Ritchie and Spencer, 1994) was used to analyze the interview data, assisted by NVivo 10 software. Analyses were guided by the following research questions: (1) did the bag use in England differ between T1 and T2, and how this was articulated by the participants; (2) did the attitudes to the English PBC differ between T1 and T2, and how this was explained by the participants; (3) did the attitudes to other similar environmental charges differ between T1 and T2, and how this was pointed out by the participants.

Data analysis was conducted in four steps. (1) All transcripts were read and pre-coded by one author (ES). This initial process resulted in the definition of codes related to the main topics (see above) as well as to new, emergent themes. An analysis of this pre-coding and code rearrangements were discussed between three authors (ES, WP, and GT). (2) Transcripts were fully coded by one author (ES), and then independently checked by another author (EW). Consensus over the diverging items was reached between the two authors through discussion, and categories refined. (3) Codes were abstracted, and the key themes mutually agreed between WP and ES. (4) The key themes were presented in detail to the rest of the team, discussed between them, and the necessary changes were made. Throughout the analysis, the interpretation was compared with the verbatim data. Direct anonymised quotations from the interviews are used in this paper in order to illustrate the key themes and sub-themes. Participant’s gender, age category, country, and time points of the study are indicated for each quotation.

### Longitudinal Observational Study

For the longitudinal observation study, we observed bag use among shoppers as they exited supermarkets in two mid-sized cities in England and Wales in July 2015 (*n* = 1,637) and July 2016 (*n* = 2,127). The study was approved by the Welsh School of Architecture Research Ethics Committee (EC1506.237). The observations were conducted at four different supermarket stores of different size, location, and prestige, with comparable stores matched in England and Wales: (1) a local branch of a mid-range supermarket brand located in the city center, (2) a budget supermarket brand located on the outskirts of a city center, (3) a mid-range supermarket brand located on the outskirts of a city center, and (4) a premium supermarket brand located on the outskirts of a city center. All of these supermarket brands provided single-use plastic bags for free prior to the introduction of the bag charge in England.

Observations for each store took place at three time points: a weekday between 10:30 and 11:30, a weekday between 16:30 and 17:30, and on a Saturday either at 11:00–12:00 or at 13:00–14:00. Observations were conducted between June 25 and July 25, 2015, when the Welsh carrier bag charge was already in effect but the English plastic bag charge was not, and again between June 22 and July 23, 2016, when both charges were in effect. Observations were conducted by one of the authors (EW), assisted by a second trained observer.

Supermarket brand status was derived from YouGov Profiles (YouGov, 2016), a market research company using data from a survey panel representative of Great Britain. YouGov Profiles provides data on characteristics of shoppers who visit supermarket chains, including the proportion of those using supermarkets who fit the National Readership Survey (NRS) social grade of ABC1 (Upper and upper middle class) and those of C2DE social grade (working and non-working class). Compared to 53% of the United Kingdom population classified as ABC1 social grade, 46% of Budget Supermarket shoppers were ABC1, 58% of Mid-range supermarket shoppers were ABC1, and 73% of Premium supermarket shoppers were ABC1.

A total of 3,764 shoppers were observed: 1,961 in Wales (818 in 2015 and 1,143 in 2016), and 1,803 in England (819 in 2015 and 984 in 2016). Two researchers located outside of stores observed all shoppers exiting the supermarkets at the different time slots. Researchers then recorded the type and number of bags used, as well as the age, gender, and group size of the observed shoppers (i.e., shopping alone, as a couple, etc.). Inter-rater reliability for recording bag use was high (all Cohen’s κ > 0.75), with differences resolved through discussion between the two observers.

## Results

Full sets of statistical analyses can be in found in the Supplementary Information.

### Changes in Behavior

**FIGURE 1:**
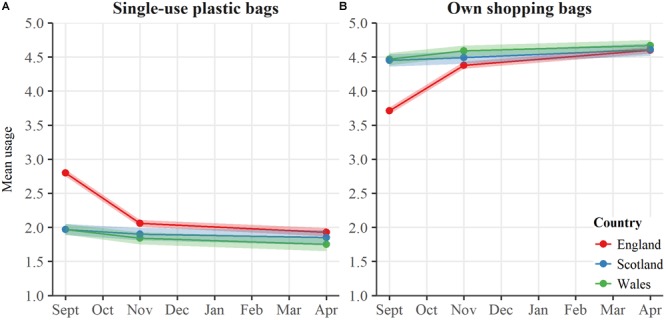
Estimated marginal means for frequency of bag use on food shopping trips. **(A)** Frequency of using single-use plastic bags and **(B)** frequency of using own shopping bags. Shaded bars indicate 95% confidence intervals.

Full details of behavior change can be found in Sections 2.1 and 2.2 of the Supplementary Information. Survey data indicated that frequency of plastic bag use in England fell substantially after the plastic bag charge was introduced (see Figure 1A), corroborating previous research (Poortinga et al., 2013; WRAP, 2015; zero Waste Scotland, 2015; Thomas et al., 2016). For frequency of taking plastic bags, the fixed effect of Country was significant, [*F*(2,2576.76) = 72.28, *p* < 0.001], as well as the fixed effect of Time, [*F*(2,1731.31) = 116.13, *p* < 0.001], demonstrating that frequency of behavior significantly varied over time and between countries. A significant interaction between Time and Country was observed [*F*(4,1731.46) = 62.49, *p* < 0.001], indicating that frequency of plastic bag use varied over time between countries. Respondents in England reported an immediate reduction in plastic bag use after the charge was introduced, with further significant reductions between 1 and 6 months after the charge. Accordingly, the frequency of taking own shopping bags continuously increased among respondents in England over the course of the survey (Figure 1B). The fixed effect of Country was significant for frequency of using own shopping bags, [*F*(2,2722.16) = 52.14, *p* < 0.001], as was the fixed effect of Time, [*F*(2,1876.67) = 132.99, *p* < 0.001], demonstrating that frequency of own bag use significantly varied between countries and over time. A significant interaction between Time and Country was also found [*F*(4,1876.29) = 65.62, *p* < 0.001], indicating that change in frequency of own bag use over time varied between the countries. Six months after the plastic bag charge was introduced, plastic bag use and own bag use in England was statistically indistinguishable from Wales and Scotland where bag charges were introduced in 2011 and 2014, respectively, indicating a quick response to the English plastic bag charge, a consistency of effects of bag charges across countries, and a lasting influence of similar policies in Wales and Scotland.

**FIGURE 2:**
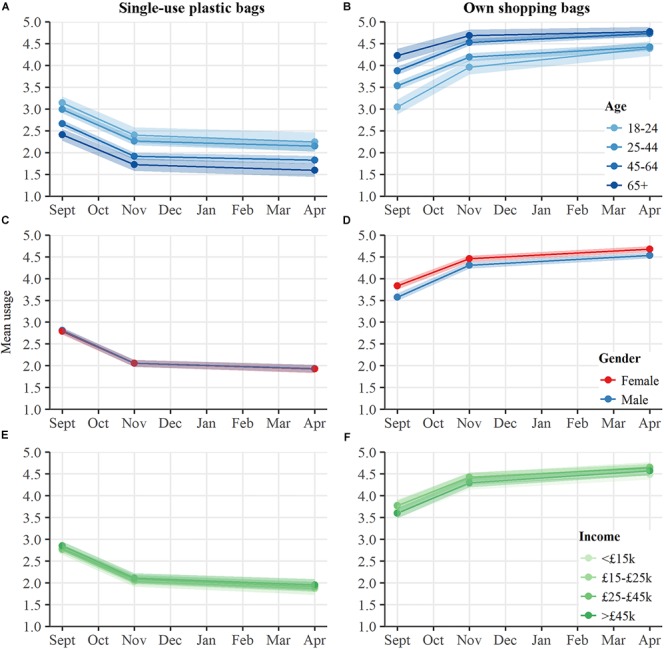
Reported frequency of plastic bag use, and use of own shopping bags, when food shopping. Responses shown for sample in England, broken down by Age group **(A,B)**, Gender **(C,D)**, and Annual Income **(E,F)**. Shaded bars indicate 95% confidence intervals.

We then compared bag use in England across demographic and socio-economic groups to determine how they responded to the plastic bag charge. As seen in Figure 2, younger respondents were significantly more likely to use plastic bags [significant fixed effect of Age, *F*(3,1491.05) = 39.44, *p* < 0.001], and less likely to take their own shopping bags [significant fixed effect of Age, *F*(3,1615.84) = 45.56, *p* < 0.001], while men were less likely to use own shopping bags than women [significant fixed effect of Gender, *F*(3,1615.84) = 45.56, *p* < 0.001], with *post hoc* comparisons (Šidák corrected) indicating that in general, Men (*M* = 4.14, SE = 0.03) had lower use of own bags than women (*M* = 4.32, SE = 0.03), (M_diff_ = -0.18, *p* < 0.001). However, interaction effects between time and demographic groups indicate no evidence that the change in plastic bag use over time varied significantly between groups: all gender, age, and income groups reduced their use of plastic bags at a similar rate. Similarly, we found no evidence that the change in use of own shopping bags across time varied across the different demographic or socio-economic groups, despite some initial differences.

The behavior change identified in the survey was corroborated by the observational field study of shoppers’ use of bags as they exited stores pre and post-bag charge. Table 3 shows just over a half (55%) of shoppers in England used plastic bags prior to the bag charge, falling to one in five shoppers (21%) after the charge was introduced. Formal analyses indicate in Wales (where a bag charge was introduced in 2011) bag use remained stable over time, and similar to behavior observed in England 9 months after the English bag charge was introduced.

**Table 3:** Proportion of shoppers classified by their observed use of bags when exiting stores in Cardiff (Wales) and Bristol (England) in July of 2015 and 2016.

	Wales		England	
	2015	2016	2015	2016
Only plastic bags	13%	14%	48%	17%
Plastic and reusable bags	4%	4%	7%	4%
Only reusable bags	53%	56%	21%	53%
Other containers	10%	11%	15%	18%
No bags observed	19%	16%	10%	8%
N (observations)	818	1143	819	984

*Total number of observations within each country for 2015 or 2016 also shown.*

We collected observational data in England across supermarkets varying in typical socio-demographic shoppers, described here as budget, mid-range, and premium supermarket stores, as well as a smaller local store of the mid-range supermarket brand. Comparisons of bag use pre- and post-bag charge (Table 4) again demonstrate that behavior change occurred across the supermarket range in England, with no indication that plastic bag use was higher at stores that typically attract more affluent shoppers.

**Table 4:** Proportion of observed shoppers in England using types of shopping bags, separated by socio-economic profile of supermarket store.

	Local		Budget		Mid-range		Premium	
	2015	2016	2015	2016	2015	2016	2015	2016
Only single-use plastic bags	56%	25%	41%	11%	51%	18%	44%	14%
Single-use plastic and reusable bags	4%	5%	8%	2%	9%	5%	5%	5%
Only reusable bags	11%	33%	14%	57%	26%	64%	31%	59%
Other containers	22%	30%	22%	26%	8%	5%	8%	9%
No bags observed	8%	7%	14%	11%	7%	18%	12%	14%
N (observations)	203	236	208	260	208	249	200	239

*Total number of observations at each supermarket for 2015 or 2016 also shown.*

Findings from the interview study corroborate survey and observational data that indicate major behavior change following the introduction of the charge, and show how people in England themselves articulated these changes. In particular, interview data demonstrates how participants have experienced the charge as a catalyst for reducing the strength and automaticity of the single-use bag use habit. These changes in behavior occurred regardless of age, gender or income as all interview participants in England reduced or completely stopped single-use bag use.

After the charge was introduced in England, participants referred to the formation of a new habit of bringing own bags to stores: “*I have remembered* [to bring] *my bags a lot more now”* (Female, 31-40, England, T2); *“We’re getting into the habit now of taking our own bags with us”* (Male, 51-60, England, T2). Participants mentioned that the charge has made them think and plan on using their own bags, instead of wasting plastic bags: “[The bag charge] *makes people think ahead and plan, and not just take things for granted*” (Female, 31-40, England, T2), *“It makes me aware of the fact that I’m paying for something that I’m only going to use for a few minutes”* (Male, 31-40, England, T2).

A large majority of interview participants in England found that they could change their behavior quickly and easily in response to the charge introduction: *“It’s very easy to carry* [your] *own shopping bags”* (Male, 51-60, England, T2), *“I think it’s* [the introduction of the charge] *gone reasonably smoothly”* (Male, 51-60, England, T2). Interview findings from Scotland and Wales equally show that adaptation to plastic bag charges in these countries was quick and effortless: *“Probably just a couple of weeks, once you got used to it, it didn’t take long”* (Female, 20–30, Scotland, T1).

Interview data demonstrates how particular social practices were developed and sustained for this behavior change to be supported. For example, some female participants mentioned carrying pouch bags in their handbags: *“If I buy something on a whim, I have one of those little fold up ones* [bags] *that goes in my handbag”* (Female, 31–40, England, T2). The majority of people with vehicles adopted the new routine of storing reusable bags in their cars: *“It’s part of my routine now. I do my food shop, I come in the house, empty the bags out, put all the food away, and before I forget, I get hold of the bags, and put them back in the car, so I know then, next time, if I need to get any shopping, I’ve got my reusable bags in the car already”* (Male, 41–50, England, T2).

### Changes in Support

**FIGURE 3:**
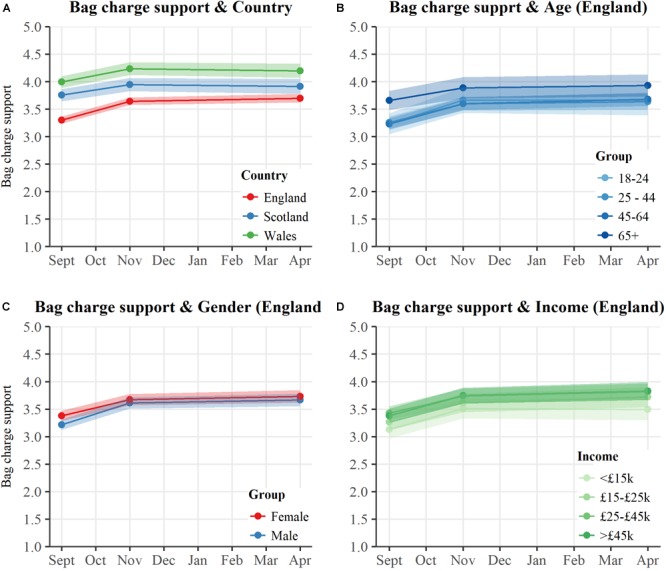
Strength of support for a 5-pence charge to consumers for each plastic bag taken over an 8-month period from September 2015. Shaded bars indicate 95% confidence intervals. **(A)** Bag charge support over time between respondents in England, Scotland, and Wales. **(B)** Bag charge support among respondents in England, separated by age group. **(C)** Bag charge support among respondents in England, separated by gender. **(D)** Bag charge support among respondents in England, separated by annual income group.

Full details of bag charge policy support change can be found in Sections 2.3 and 2.4 of the Supplementary Information. Analyzing survey respondents’ attitudes toward the bag charge, we find that support for a five pence bag charge increased the month after the English bag charge was introduced (Figure 3A), which is in line with previous findings (Convery et al., 2007; Poortinga et al., 2013). A significant fixed effect for Country [*F*(2,2898.44) = 53.60, *p* < 0.001] and a significant fixed effect of Time were observed, [*F*(2,1616.76) = 56.93, *p* < 0.001], demonstrating that support for a bag charge significantly varied between countries and over time. Analysis indicated a significant interaction between fixed effects of Country and Time for support for a plastic bag charge [*F*(4,1617.98) = 4.20, *p* = 0.002], where changes over time in bag charge support varied between countries. Prior to the English charge, public support was higher in Wales and Scotland where charges were already in place, but support also increased in these countries 1 month after the English charge was introduced. Šidák corrected *post hoc* comparisons showed plastic bag charge support grew between T1 and T2 in Wales (*M*_diff_ = 0.24, *p* < 0.001) and in Scotland (*M*_diff_ = 0.19, *p* = 0.001), but did not significantly change between T2 and T3 for Wales (*M*_diff_ = 0.04, *p* = 0.839) or for Scotland (*M*_diff_ = 0.04, *p* = 0.906).

Comparing policy support across demographics, some general differences in bag charge support can be seen, with younger people generally less supportive of a bag charge; significant fixed effect of Age, [*F*(3,1729.44) = 4.16, *p* = 0.006]. Yet as seen with the analysis of frequency of bag use, interaction terms between demographic group and change in support over time show no significant effects for variation in how support for a bag charge changed over time among gender [*F*(2,960.22) = 1.50, *p* = 0.225], age [*F*(6,981.12) = 0.65, *p* = 0.687], or income groups [*F*(8,349.88) = 0.49, *p* = 0.860] (Figure 3B–D). All demographics increased their support for the policy in the 1-month period after the charge was introduced, with no significant changes between one and 6 months post charge.

These changes in policy support were corroborated by interview findings that indicated an increase in the level of support for the charge in England, with all interview participants expressing positive views after the charge was introduced, regardless of age, gender or socio-economic status. In particular, there was an understanding of environmental benefits of the charge. People in England spoke about the charge being an effective policy instrument to reduce plastic bag waste and raise environmental awareness: “*I don’t think it’s a bad idea. It definitely encourages people to either buy a reusable bag, or use other things to put them in, or just not take one at all if you’ve only got a couple of items”* (Female, 31–40, England, T2), *“I think it’s a good idea, I’ve seen more people taking their* [own] *bags to the shop, so less is getting wasted”* (Male, 20–30, England, T2), *“I’m glad there’s a charge on plastic bags because we need to do something. I would hope that it is going to make a difference to landfill and to the way people think in general about the things that they dispose of”* (Female, 51–60, England, T2). Support for the plastic bag charge was equally high in Wales and Scotland, where it was also recognized as an effective environmental policy instrument: *“I’ve been in total agreement with it* [bag charge] *for years before it came in, and I always thought it would have been a good idea to do it. So I was pleased when they did introduce that. Statistically, I’ve seen it on the news, that it’s cut down the number of bags that we waste”* (Male, 41–50, Wales, T1).

### Policy Spillover

With increased support for the bag charge in England, we investigated policy spillover, whereby people who increased their support for the bag charge may also increase their support for other environmental policies. The longitudinal survey measured support for three hypothetical policies: a five pence charge on plastic bottles, a five pence charge on items with excessive packaging, and higher tax on fuel for environmental reasons (Descriptive statistics for support for each policy by country and time can be found in section 2.5 of the Supplementary Information). Multiple regression analyses modeled how the change in support for the plastic bag charge predicted changes in support for each hypothetical policy (Table 5). Results show that among respondents in England, those who increased their support for the plastic bag charge were more likely to report increased support for two additional policies: a charge for plastic bottles and a charge for excessive packaging. The positive links between greater bag charge support and increased support for other waste-reduction policies were consistent between 1-month changes in policy support (between T1 and T2), and lasting changes in support (6 months between T1 and T3).

**Table 5:** Summary of linear regressions predicting change in support for hypothetical policies of a charge for plastic water bottles (ΔWater Bottle), charge for excessive packaging (ΔPackaging), or higher fuel duty (ΔFuel Duty), as predicted by change in support for the plastic bag charge (ΔBag Charge).

Timeframe	Outcome	Coefficient	*B*	SE	Beta	Sig	CI	*N*
T1 to T2	ΔWater Bottle	*Constant*	*0.83*	*0.08*	*0.00*	*<0.001*	*0.68: 0.98*	1124
		ΔBag Charge	0.23	0.03	0.22	<0.001	0.18: 0.29	
	ΔPackaging	*Constant*	0.96	0.08	0.00	*<0.001*	0.81: 1.11	1133
		ΔBag Charge	0.22	0.03	0.20	<0.001	0.16: 0.28	
	ΔFuel Duty	*Constant*	0.59	0.05	0.00	*<0.001*	0.49: 0.69	1132
		ΔBag Charge	0.01	0.02	0.02	0.586	–0.03: 0.06	
T1 to T3	ΔWater Bottle	*Constant*	0.95	0.09	0.00	*<0.001*	0.77: 1.13	695
		ΔBag Charge	0.22	0.04	0.21	<0.001	0.15: 0.29	
	ΔPackaging	*Constant*	0.99	0.10	0.00	*<0.001*	0.80: 1.18	698
		ΔBag Charge	0.20	0.04	0.17	<0.001	0.12: 0.27	
	ΔFuel Duty	*Constant*	0.69	0.07	0.00	*<0.001*	0.56: 0.83	703
		ΔBag Charge	0.03	0.03	0.03	0.340	–0.03: 0.09	

*Regressions include changes between T1 and T2, and between T1 and T3. All regressions included covariate of baseline support (T1) for hypothetical policies to control for regression to the mean effects.*

Interviews in England also addressed support for the same three hypothetical policies, and, once more, corroborated the survey findings. For the packaging-related policies, participants came to support these policies at T2, highlighting the values of the perceived need to reduce plastic waste and raise environmental awareness: *“I’m very aware of the amount of plastic bottles, so yeah, I think if that* [charge] *came in, it would make me think about what I was buying”* (Female, 41-50, England, T2), *“If you want to buy four apples and they come in a foam type dish, and then that’s wrapped in plastic, I think that needs to be addressed. I don’t think there’s any need for all that plastic”* (Female, 20-30, England, T2).

We found no link in the survey data between changes in support for the bag charge and changes in support for increased fuel duties for environmental reasons. This suggests a limit to policy spillover effects, where people view other nominal fees to customers to reduce waste more favorably after a bag charge, but with no significant changes in views for less similar charges, despite having similar pro-environmental motives. Interview data also reflected low support for fuel duties rise, further highlighting that such policy would affect those on lower income and businesses: *“Well, the fuel charges, that affects everybody doesn’t it: businesses, pensioners who only use their car once a week, so I don’t think I’m in favor of that if it’s right across the board”* (Female, 51-60, England, T2). Participants also suggested that instead of fuel duties rise, governments should seek more sustainable alternatives: *“I think instead of just putting charges on things, they should be looking more into utilizing renewable sources of energy, cleaner cars, things like that, that I think is better in the long run”* (Male, 20-30, England, T2).

## Discussion

Policies enforcing a charge to customers for plastic bags have been implemented worldwide (Miller, 2012), and five pence charges on single-use bags have produced large changes in the wholesale volume of bags issued in Wales, Northern Ireland, and Scotland (Convery et al., 2007; Poortinga et al., 2013; zero Waste Scotland, 2015). Here we present a comprehensive evaluation of the English plastic bag charge introduced in October 2015, and the first longitudinal study to assess individual behaviors and attitudes immediately before and after the policy was introduced.

Results show widespread positive behavioral changes across socio-demographic groups, where single-use plastic bag use decreased and own shopping bag use increased. The observed broad compliance with the charge may be surprising given the small cost of the charge, especially given that high income groups and supermarkets of typically high-income shoppers also demonstrated significant behavior change. This suggests that a plastic bag charge is not only an economic instrument, but also a psychological one. From an economic perspective, we would expect responses to a bag charge to vary across different socio-economic groups (cf., Dikgang et al., 2012, 2012). Behavior of higher income groups would presumably be less affected by the charge as compared to lower income groups (as a five pence cost would constitute a smaller part of the household budget), and presumably lower income groups would have a lower favourability of the charge than higher income groups. Instead, the results are much more in line with a “habit disruption” perspective (Poortinga et al., 2013) in that the charge changed or “disrupted” habits regardless of financial situation. The qualitative results further support this interpretation, as shoppers reported that the charge made them reconsider their behavior, and adopt new routines.

One of the other main findings of the study is that support for the English plastic bag charge increased across the board. That is in line with previous research in Wales (Poortinga et al., 2013), as well as with studies showing similar attitudinal effects for other environmental, safety and health policies. Awareness and agreement with the policy likely explains the widespread significant increase in support for the policy just 1 month after it was introduced. Many of the interview respondents highlighted the ease of compliance with the policy, but also understood the environmental motivations behind the bag charge, and expressed widespread support for these policy goals. Together, this indicates that the bag charge did not have any adverse distributional effects, but rather was effective and supported across society and socio-economic groups.

The widespread support also appears to extend beyond bag charges, and we show what we believe to be the first evidence for policy spillover effects, whereby greater support for the bag charge predicted greater support for policies of similar size and scope. Bag charges have been largely unsuccessful at encouraging *behavioral* spillover (Poortinga et al., 2013; Thomas et al., 2016), where bag use behavior after a bag charge was introduced is not predictive of changes in other sustainable behaviors. The potential of *policy* spillover is substantial, however, given the importance of public support for creating and implementing policies (Burstein, 2003). Responses to climate change and other sustainability issues demand significant policy changes (IPCC Climate Change, 2014), and fostering public support may well embolden politicians to take stronger action. Although a bag charge may be limited in scope for tackling climate change or other consumption-related problems (e.g., resource depletion, landfilling), we show that accessible and popular policies may well foment a greater acceptance of similar policies, which may galvanize public support to additional sustainability policy action. We recognize that the policy spillover effects were not found to all policies that this study addressed. Indeed there appears to be a limit to policy-spillover effects, in that they appear to be restricted to the domain of the original policy, in this case (single-use) plastics and packaging. This is consistent with previous literature indicating behavioral spillover is more likely within than between domains (e.g., waste, transport; Thøgersen and Ölander, 2003; Whitmarsh and O’Neill, 2010) due to conceptual links being stronger between similar behaviors and/or situational barriers limiting spillover beyond a particular context. Additional work is needed to determine whether policy spillover effects can be used to strengthen public support for changing more structurally embedded unsustainable practices.

Our research also highlights the value of applying different methodologies on large-scale comparisons between the different United Kingdom countries, something that we have termed the “Devolution Lab.” Paun et al. (2016) observed that devolution in the United Kingdom (i.e., the delegation of powers from national to subnational governments) is designed to allow for policy differentiation and divergence at the sub-national level. This provides an opportunity for policy innovation, whereby different approaches can be tried and tested. Furthermore, devolvement of policy powers produces a natural-experimental structure that allows for systematic data collection with ready-made comparators. This is clearly illustrated by the carrier bag charges that were introduced at different times in Wales (2011), Northern Ireland (2013), Scotland (2014), and England (2015), with some cross-country variation in the policy (the charge in England is only for plastic bags, whereas in the other countries it has to be paid for paper bags), but can also apply to other devolved policy areas, such as education, transport, health, and social care. The Devolution Lab as a place for testing new policies as well as a research methodology to examine their effectiveness and/or behavior change theory in a “real-life” natural experiment is not only relevant to the United Kingdom, but also to other countries with devolved Governments, such as Australia, and federal states, such as the United States and Germany.

A key strength of the current study was the use of multiple research methodologies, with data being collected at multiple time points before and after the charge was introduced. In particular, the inclusion of observational data has helped to validate the findings of the longitudinal survey. Measuring pro-environmental behavior and attitudes may be prone to self-presentation biases, with the desire to appear more environmentally friendly that one behaves (Thomas and Walker, 2016). Objective measures of bags used by shoppers in a field observation give additional credence to the survey. In addition, the interviews have further corroborated these findings and provided information grounded in participants’ experiences of the charge, on how the charge may have worked, and how it changed people’s views on the policy, as well as catalyzed a wider waste awareness among the public. The findings across the different methods converges on a consistent picture of support for, and adaptation to, the bag charge. The use of surveys, interviews, and observations has enabled us to overcome limitations of single methods, such as bias in self-reports of bag reuse, and provided both depth and breadth to the analysis of a national behavior change policy. This can act as a model for evaluating other policies and or interventions aimed at changing (environmental) attitudes and behavior.

There remain several areas unaddressed here that warrant further investigation. We did not examine bag use outside the context of consumption. Reusable bags are generally beneficial over single-use plastic bags, but this depends on them being reused several times (Lewis et al., 2010; Edwards and Fry, 2011). Further research should examine how bags are reused and how bag charging may have impacted on other uses for carrier bags (e.g., lining bins). Research on bag charging is also needed over the longer term. While we explored a 7-month period here, other researchers have found some evidence of recidivism once consumers have adapted, suggesting the charge may need to be increased to maintain its “shock factor” in disrupting habits. A bag charge policy in South Africa (Dikgang et al., 2012) found plastic bag use fell once a charge was introduced, but after the charge was reduced 3 months after introduction, plastic bag usage increased over several years. The example of the Irish bag charge also suggested that bag use rose in the 6 years after the levy was introduced, and increasing the charge was linked to a further reduction in bag usage (Clarke, 2014). Although we find that bag usage in Wales remained low since their charge was introduced in 2011, further evaluation of bag charge policies is warranted to identify best practice for maintaining long term behavior change.

## Ethics Statement

This study was carried out in accordance with the recommendations of the Ethics Committee of the Welsh School of Architecture, Cardiff University, for the longitudinal survey (EC1507.239), longitudinal interviews (EC1507.243), and longitudinal observation study (EC1506.237). All subjects in the survey and interview study gave written informed consent in accordance with the Declaration of Helsinki, and the observational study was conducted in a public place.

## Author Contributions

GT designed and conducted the study, led the analysis of the longitudinal survey study, the analysis of the observational study, and writing of the paper, contributed to the design and supervision, and participated in data collection and analysis for the interview study. ES designed and conducted the study, led the analysis of the longitudinal interview study, contributed to the design, supervision, and the analysis of the observational study, contributed to the design and analysis of the survey study, led on writing of the interview study, and contributed to the writing of all other sections of this paper. WP was the principal investigator on this project and contributed to the design and analysis of all elements of the research project and writing of all sections of this paper. EW co-designed the observational study, led the fieldwork for this study and contributed to the writing of its methodology, and participated in data collection of the interview study. LW was the co-investigator on this project and contributed to the design of survey and interview studies and writing of all sections of this paper.

## Conflict of Interest Statement

The authors declare that the research was conducted in the absence of any commercial or financial relationships that could be construed as a potential conflict of interest.
